# COVID-19-Associated Intussusception Presenting Atypically With Bilious Emesis: A Case Report

**DOI:** 10.7759/cureus.105933

**Published:** 2026-03-26

**Authors:** Han O Lim, Michael J Clarion

**Affiliations:** 1 Surgery, Madigan Army Medical Center, Tacoma, USA; 2 Pediatrics, Madigan Army Medical Center, Tacoma, USA

**Keywords:** bilious emesis, bilious vomiting, covid-19, idiopathic intussusception, pediatric gi surgery

## Abstract

COVID-19 can present with gastrointestinal symptoms in infants; an uncommon presentation may be intussusception. There are rarely reported cases of intussusception due to COVID-19 in the literature. Of the reported cases, only one case presented with bilious emesis. Bilious emesis is an atypical manifestation of intussusception and is more commonly linked to malrotation. We report an additional case of COVID-19-related intussusception, which presented with bilious emesis. Although the prior case required surgical intervention, our case was treated successfully with an air enema reduction. These cases highlight bilious vomiting as a possible presentation of COVID-19-related intussusception and warrant inclusion of COVID-19 in the differential diagnosis of severe intussusception in infants.

## Introduction

While COVID-19 is largely considered a respiratory disease, it is known to have multisystemic effects. Infants are frequently asymptomatic or have only mild disease [1,2]. In this case report, we report an uncommon presentation of intussusception in the setting of SARS-CoV-2 infection, manifesting with bilious emesis. A four-month-old female had mild respiratory symptoms and was diagnosed with ileocolic intussusception requiring air enema reduction. The patient recovered fully. This case highlights bilious emesis as a rare but important symptom of COVID-19-associated intussusception and emphasizes the need to consider intussusception in infants with bilious vomiting during SARS-CoV-2 infection.

## Case presentation

**Figure 1:**
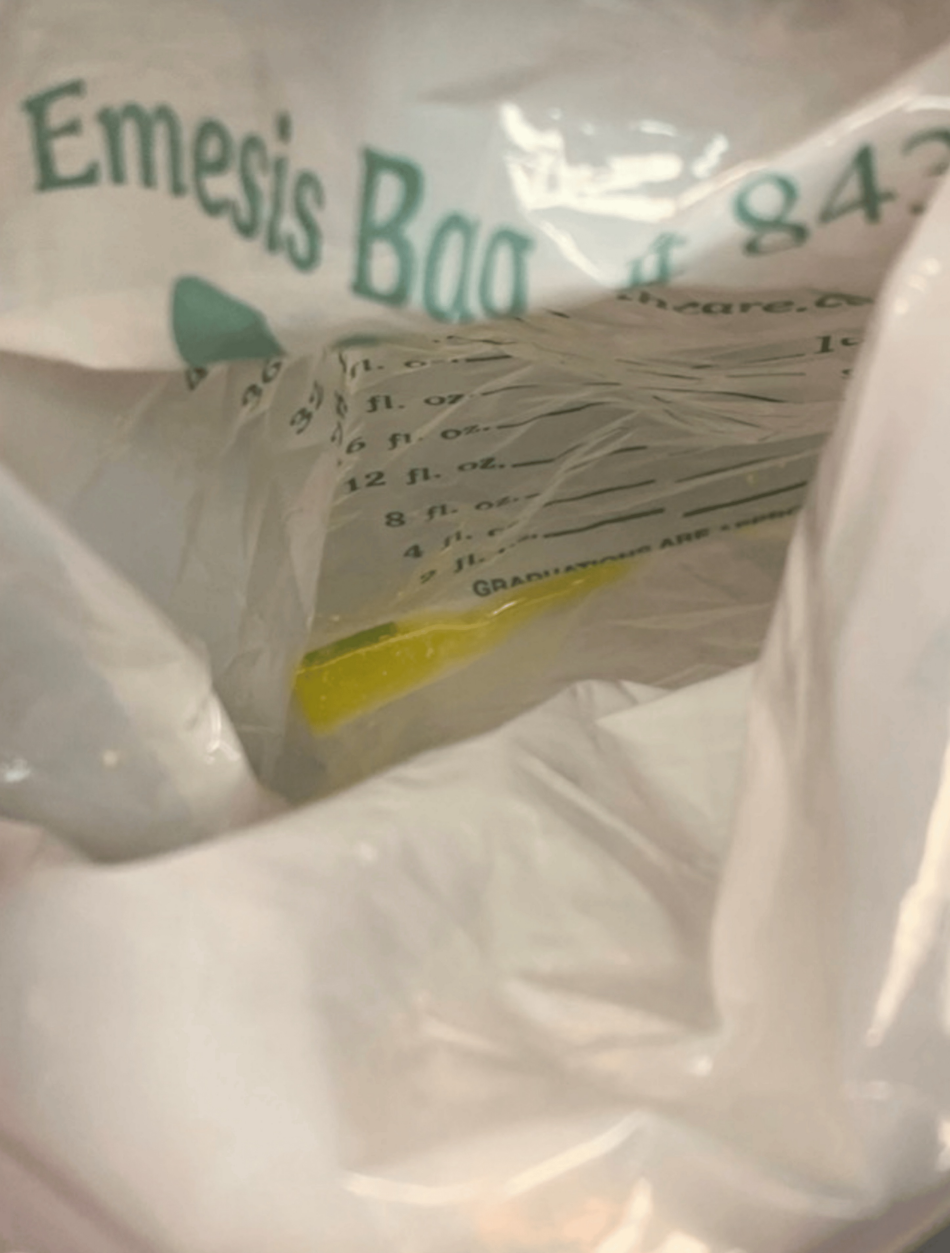
Bilious emesis collected in the exam room

A former full-term four-month-old female presented to the emergency department with one day of episodic, progressively bilious emesis. Three days before presentation, the patient developed mild congestion, cough, and intermittent fussiness. Parents denied increased work of breathing, apnea, cyanosis, diarrhea, or blood/mucous in the stool. There were no known sick contacts, although the patient was in daycare. Upon examination, the patient was well appearing, afebrile, and hemodynamically stable. She had scant clear rhinorrhea but no other respiratory abnormalities. During the exam, the patient was intermittently fussy and had an episode of small-volume bilious emesis (Figure 1).

**Figure 2:**
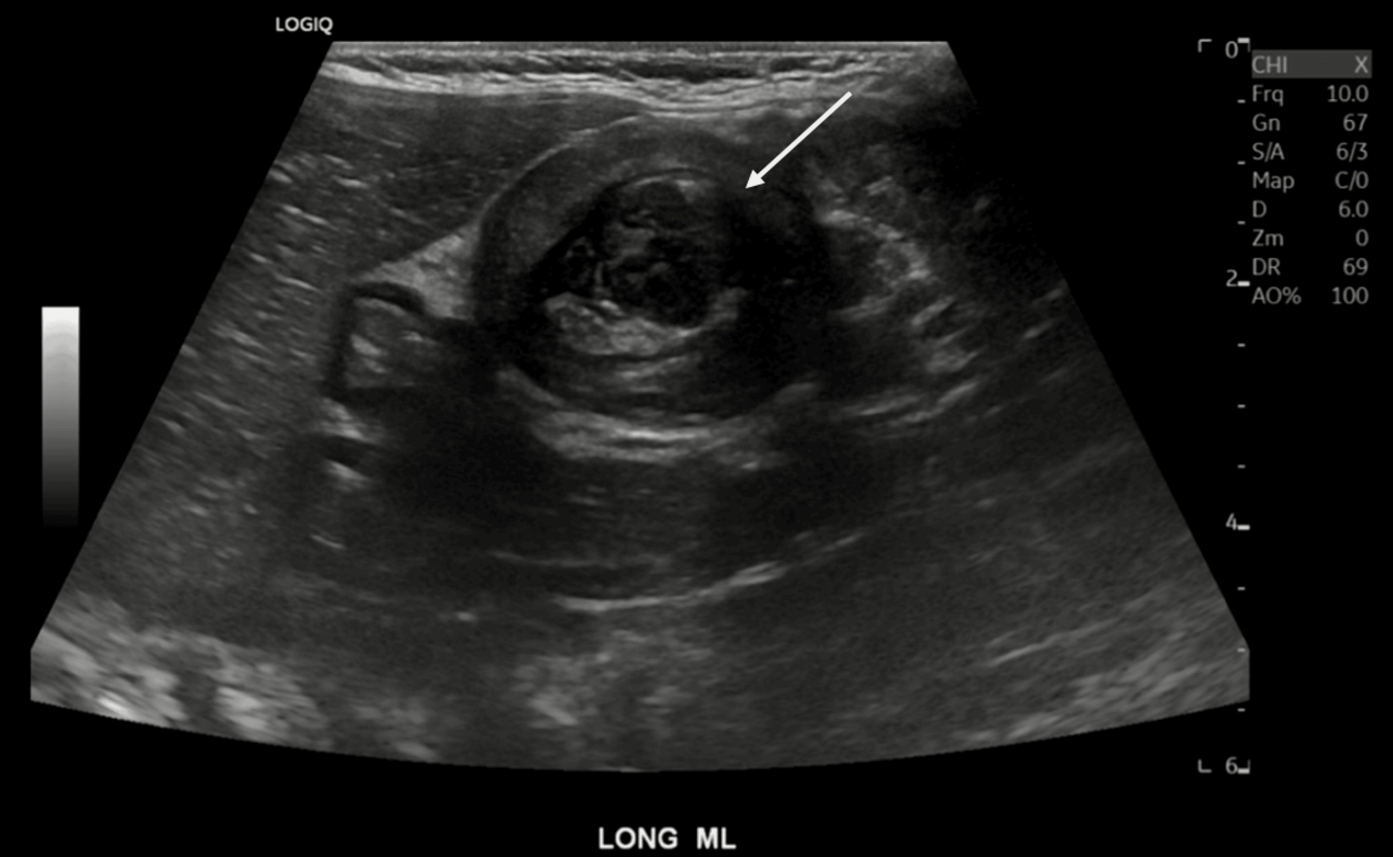
Ileocolic intussusception on abdominal ultrasound (midline long-axis view)

Evaluation included an abdominal film, which showed a non-obstructive bowel gas pattern, and a SARS-CoV-2 nasopharyngeal PCR, which was positive. Due to the bilious emesis and concern for malrotation, an upper gastrointestinal (GI) series was obtained, which was normal. The patient was admitted for observation and continued to have bilious emesis with intermittent fussiness. A repeat abdominal film showed mildly dilated right lower quadrant loops of bowel. Given the persistent symptoms and new radiographic findings, an abdominal ultrasound was performed, which demonstrated ileocolic intussusception (Figure 2). Pediatric surgery performed an air enema reduction. The patient’s fussiness and emesis subsequently resolved. The next day, a repeat ultrasound revealed mesenteric lymphadenopathy with a small amount of simple free fluid in the abdomen, consistent with a reactive process. The intussusception did not recur. Our patient tolerated oral intake and was discharged later that day.

## Discussion

Intussusception in the pediatric population is most commonly idiopathic. Nevertheless, there is evidence that viral infection can trigger intussusception via hypertrophy of intestinal lymphatic tissue, particularly in the terminal ileum. In the literature, Makrinioti et al. reported one additional case of SARS-CoV-2-associated intussusception with bilious emesis [3]. A 10-month-old female in London, United Kingdom, initially presented with bilious vomiting and red currant jelly stools after two weeks of coryzal symptoms and conjunctivitis. An air enema reduction attempt was unsuccessful, prompting surgical exploration. A Ladd's procedure was performed after intussusception and malrotation were identified. The patient recovered fully after the procedure [3]. As SARS-CoV-2 is known to infect the GI tract and can result in mesenteric lymphadenitis, it is likely that acute SARS-CoV-2 infection was the cause of the intussusception in both patients [4-6].

Vomiting is a frequent symptom of intussusception, but bilious emesis is uncommon. In most reported series, vomiting is non-bilious, with colicky abdominal pain and bloody stools being more characteristic features [4-6]. Bilious vomiting occurs due to obstruction distal to the ampulla of Vater and, in infants, raises suspicion for malrotation with midgut volvulus. In our case, this prompted an upper GI series to exclude malrotation. In the case reported by Makrinioti et al., a malrotation was indeed found intraoperatively [3]. These findings illustrate how bilious emesis may mislead clinicians and potentially delay the diagnosis of intussusception.

These two cases add to the growing literature describing intussusception in the setting of COVID-19 [3-6]. What distinguishes them is the shared presentation of bilious vomiting, a rare but important symptom that expands the clinical spectrum of COVID-19-associated intussusception. Although the mechanism for this presentation is unclear, it may reflect more proximal obstruction or delayed recognition due to atypical symptoms.

## Conclusions

Bilious vomiting in infants usually raises concern for malrotation with volvulus or other bowel obstruction distal to the ampulla of Vater. Our case and a previously reported case in the literature demonstrate that intussusception, particularly in the setting of SARS-CoV-2 infection, can also present with bilious emesis. Our patient recovered fully with appropriate management. However, clinicians should maintain a high index of suspicion and pursue early abdominal ultrasound in COVID-positive infants with bilious vomiting to avoid diagnostic delay.
